# The effect of magnetic resonance image slice thickness and voxel geometry on digital dental implant planning: an in vitro study

**DOI:** 10.1007/s10006-026-01662-1

**Published:** 2026-11-10

**Authors:** Hian Parize, Fábio Munhoz, Carlos Cordeiro, Norbert Meier, Johannes Kleinheinz, Dalva Cruz Laganá, Newton Sesma, Lauren Bohner

**Affiliations:** 1 Department of Cranio-Maxillofacial Surgery, University Hospital Muenster, Muenster, Germany; 2 Department of Prosthodontics, University of São Paulo, São Paulo, Brazil; 3 University Hospital Muenster, Muenster, Germany; 4 Department of Dentistry, Federal University of Santa Catarina, Florianopolis, Brazil; 5 Department of Prosthodontics, University of Sao Paulo School of Dentistry, 2227 Professor Lineu Prestes Avenue, Sao Paulo, SP Brazil

**Keywords:** Dental implants, Computer-aided surgery, Magnetic resonance imaging, Anatomy, Oral and maxillofacial surgery

## Abstract

**Objectives:**

To evaluate the effect of magnetic resonance imaging (MRI) slice thickness and voxel geometry on digital dental implant planning compared to cone-beam computed tomography (CBCT).

**Methods:**

Edentulous phantoms were scanned using CBCT and two MRI protocols: anisotropic and isotropic. Two calibrated examiners digitally planned the position of four implants for each sample (*n* = 8) per exam (CBCT, anisotropic and isotropic). Diagnostic accuracy was assessed using implant angular deviation and linear measurements. Image quality was evaluated using a questionnaire regarding visualization of anatomical structures. Cost-to-planning-time ratio was assessed by exam costs per minute of planning. The Friedman and Wilcoxon tests were used to evaluate differences in diagnostic accuracy, planning time, and cost-to-planning-time ratio. Image quality ratings on a 5-point scale were classified as non-valid (≤ 2) or valid (≥ 3) for clinical use. Reliability was assessed by percentage agreement, and differences were evaluated using the chi-square test.

**Results:**

A total of 96 implants were planned, with 32 in each exam. No significant differences were observed between exams for angular deviation (*p* > 0.197), linear measurements (*p* > 0.305), image quality (*p* > 0.132), or planning time (*p* = 0.093). However, CBCT had lower cost-to-planning-time ratio than anisotropic and isotropic MRI (*p* = 0.012), which did not differ from each other (*p* = 1.000). Inter-examiner agreement on image quality for anisotropic and isotropic MRI varied widely, with lower ratings for the mandibular nerve canal.

**Conclusion:**

No statistically significant differences were detected among the imaging protocols in diagnostic accuracy or image quality under the investigated in vitro conditions.

## Introduction

The integration of digital technologies into implant dentistry has transformed the field, providing clinicians with detailed three-dimensional (3D) views of surgical sites and enabling virtual surgical simulations [1]. These advancements have improved diagnostic accuracy, leading to safer, more precise, and minimally invasive treatments—particularly for fully edentulous patients [2]. In implant planning, diagnostic accuracy refers to the trueness and precision in determining the feasibility, ideal location, and required dimensions for dental implant placement.

Cone beam computed tomography (CBCT) has traditionally been considered the reference standard imaging exam for 3D dental implant planning. However, recent advancements in magnetic resonance imaging (MRI) indicate that it may be a viable alternative, offering advantages such as no radiation exposure and improved soft tissue visualization. This can enhance the registration of surface scan data, especially in edentulous cases [3, 4]. The versatility, safety, and capability of MRI to image both structure and function have established it as a prominent tool in modern medicine, significantly impacting patient care across several specialties [5]. For example, MRI can aid in the early diagnosis of peri-implant disease by detecting osseous edema [6].

Despite its potential, the visualization and measurement of maxillofacial hard tissues via MRI require specific pulse sequence parameters due to the very short transverse relaxation times of these tissues, which result in a low detectable signal [7]. In addition, integrating MRI into daily dental practice presents significant challenges due to the complexity of oral anatomy, susceptibility to metal and motion artifacts, and high cost. Nevertheless, these obstacles have been addressed through the development of specialized MRI protocols and equipment tailored for dental imaging (i.e., dental-dedicated MRI), making MRI a more accessible and practical tool for dental professionals [8].

Initial studies of MRI-guided dental implant planning in small groups of edentulous patients and subsequent in vitro studies on human cadaver samples demonstrated that MRI-based workflows can achieve accuracies comparable to those of traditional CBCT methods [4, 9]. Previous research has evaluated the use of MRI for dental implant planning, including linear measurements and visualization of relevant anatomical structures [3]. However, further studies are still needed to fully establish the feasibility and accuracy of MRI in dental implant planning [3].

Fast field echo (FFE) is a gradient echo sequence used for dental implant planning, with nomenclature varying by manufacturer. For example, Philips Healthcare uses T1-weighted fast field echo (T1FFE), Siemens Healthineers uses fast low-angle shot (FLASH), and GE Healthcare uses spoiled gradient recalled echo (SPGR) [10]. One study found that the low signal-to-noise ratio (SNR) and poor image contrast of the T1FFE sequence limited its usefulness for differentiating cortical bone from soft tissue during single-implant planning in patients [11]. In contrast, another study reported comparable results between FLASH and CBCT for fully guided dental implant placement in hemimandibles, including both partially and fully edentulous scenarios [4]. A different study revealed that the angular deviation of implants planned in the anterior region of the edentulous mandible with FFE was significantly different from those planned using CBCT [12].

Previous studies have shown that a voxel size of 0.3 to 0.4 mm is sufficient to obtain CBCT images of acceptable diagnostic quality for implant treatment planning [13]. In MRI, the optimal voxel size is partly determined by the machine’s SNR. Higher SNR allows for finer voxel sizes and improved spatial resolution [14]. However, SNR itself is affected by several parameters, including the acquisition matrix, voxel size, field of view, surface coil type, slice thickness, echo time, and the number of signal acquisitions [15, 16]. In addition, optimization of these parameters to increase SNR may come at the expense of image resolution [17]. However, the clarity and detail of the images are determined by several parameters and factors, including the resolution level, which can affect the detection and characterization of anatomical structures [18].

In 3D imaging exams (e.g., CBCT and MRI), the voxel geometry describes the relationship between the in-plane resolution (x- and y-dimensions) and the slice thickness (z-dimension) [19]. Isotropic voxels have equal dimensions in all three directions, allowing reformatting in any plane, such as the cross-sectional and panoramic views used in implant planning, with consistent spatial resolution [19]. CBCT is typically reconstructed with isotropic voxels, whereas MRI can be acquired with either geometry. Anisotropic MRI acquisitions, in which slice thickness exceeds in-plane resolution, have historically been used to achieve adequate signal-to-noise ratio within clinically feasible acquisition times; however, they yield lower-quality multiplanar reformats and greater partial volume averaging, which may obscure small or subtle structures [20]. Voxel geometry may therefore affect the delineation of the cortical plates and mandibular canal during digital implant planning.

In addition, cost and time are critical factors in the adoption of new technologies in patient care and should be considered during the diagnostic, planning, and execution phases. In Europe, running a dental practice typically costs approximately €300 per hour, or about €5 per minute [21]. The cost per minute associated with digital dental implant planning depends on factors such as software costs, clinician expertise, workflow integration, and the use of technologies like CAD/CAM systems and 3D imaging. These technologies offer benefits, including increased precision, reduced surgery time, and fewer complications. However, comprehensive assessment of cost-to-planning-time ratio for digital implant planning remains lacking in the literature [22, 23].

Few studies have investigated the effect of MRI slice thickness and voxel geometry on dental implant planning. Therefore, the aim of this study was to compare anisotropic and isotropic 3D T1FFE MRI protocols with CBCT in terms of diagnostic accuracy, image quality, and cost-to-planning-time ratio for dental implant planning.

## Methods

**Fig. 1:**
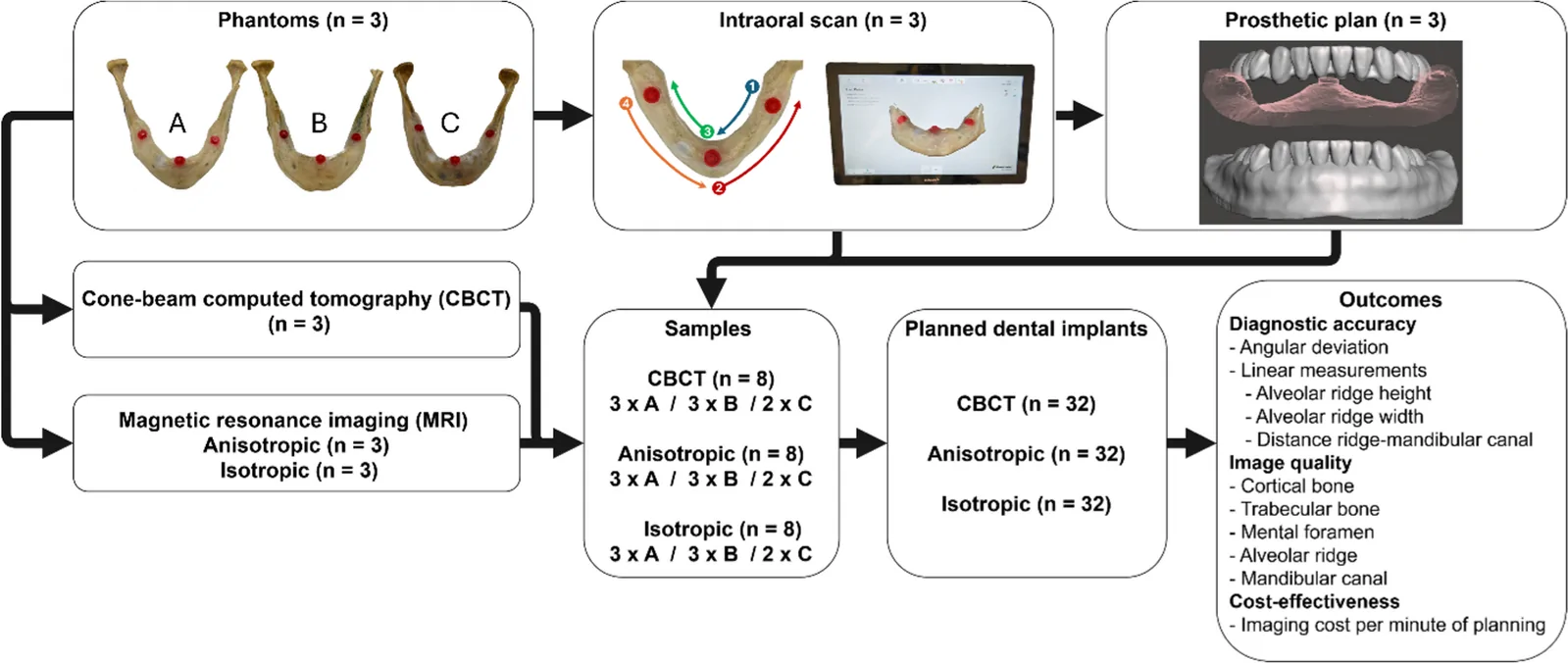
Study design

This study was reported according to the CRIS Guidelines (Checklist for Reporting In vitro Studies) [24]. The procedures for acquiring the phantoms and samples, and for measuring the angular deviation of the dental implants, taking linear measurements, and assessing image quality, have been described previously [12, 25]. The study design is represented in Fig. 1.

### Sample size calculation

The sample size was calculated to detect a mean difference of 1.0° ± 0.8° in dental implant angular deviation (primary outcome) between CBCT- and MRI-based planning, as reported in a previous study using phantom [12], in which the mean value was derived from groups of four implants planned for the rehabilitation of edentulous mandibles. A paired t-test with 80% power and a significance level of *p* < 0.05 indicated that eight samples (*n* = 8) would be sufficient (G*Power version 3.1.9.7, University of Duesseldorf).

### Phantoms and prosthetic planning

Three dry human mandibles were used to fabricate phantoms with artificial soft tissue composed of 2-mm-thick sanitary silicone (Fischer, Germany) and polymeric fiducial landmarks (myBrick, Germany). This study was conducted in accordance with the Declaration of Helsinki and received ethical approval No. 7.681.781 from the University of São Paulo. The alveolar ridges of the phantoms were digitized using an intraoral scanner (TRIOS 3, 3Shape, Denmark) according to the manufacturer’s instructions, and the resulting data were exported as standard tessellation language (STL) files. Prosthetic plans were digitally designed as removable complete denture prostheses using software (Meshmixer, Autodesk, United States) and exported as STL files. For imaging with MRI and CBCT, the phantoms were secured using dental wax (White Periphery Wax, Kemdent, England) in a container filled with water to simulate soft tissue.

### Cone beam computed tomography

The phantoms were scanned with dental tomography (Veraview x800, Morita, Japan) and standard parameters (field of view 150 × 75 mm; kV 100; mA 6; exposure 17.86 s; isotropic voxel size 0.25 mm).

### Magnetic resonance imaging

In addition to water, less than 10 mL of liquid handwash detergent (Sensiva, Schuelke, Germany) and the gadolinium contrast agent (Omniscan 0.5 mmol/mL, GE Healthcare, USA) were mixed with the phantoms in the plastic container to optimize the intensity of voxels. The phantoms were then scanned using a 3-Tesla MRI system (Ingenia CX, R5 V6.a, Philips Healthcare, Netherlands) equipped with a 32-channel head coil. Two imaging protocols were employed, anisotropic and isotropic. Both protocols used a T1-weighted 3D FFE sequence with the same flip angle. The anisotropic protocol prioritized in-plane resolution and acquisition speed, whereas the isotropic protocol provided uniform resolution in all directions at the cost of a threefold longer acquisition. The protocols were designed to contrast anisotropic and isotropic voxel geometry; to achieve each target geometry while maintaining clinically appropriate signal-to-noise ratio and image contrast, the remaining acquisition parameters (repetition time/echo time [TR/TE], pixel bandwidth, number of averages, and acquisition matrix) were co-adjusted accordingly (Table 1).

**Table 1:** MRI pulse sequence parameters

Parameter	Anisotropic	Isotropic
Sequence type	T1W 3D FFE	T1W 3D FFE
TR/TE (ms)	4.3/1.4	6.5/2
Pixel bandwidth	651	404
Number of slices	640	1024
Slice thickness (mm)	0.50	0.30
Spacing between slices (mm)	0.25	0.15
Pixel spacing (mm)	0.25	0.30
Flip angle	10°	10°
Number of averages	2	3
Number of phase encoding steps	160	130
Acquisition matrix	320/160	516/130
Voxel size (mm)	0.25 × 0.25 × 0.50	0.30 × 0.30 × 0.30
Voxel volume (mm³)	0.031	0.027
Scanning duration (min)	3	9

*TR * repetition time; *TE * echo time; *T1 3D FFE*  T1-weighted three-dimensional fast field echo

### Samples

Each sample was a “virtual patient” generated with dedicated dental implant planning software (coDiagnostiX, v. 10.5.0, Dental Wings, Inc., Canada). The three phantoms were each scanned once with the three exams: CBCT, anisotropic-MRI isotropic-MRI. The Digital Imaging and Communications in Medicine (DICOM) files from two phantoms produced three samples per exam, while the third phantom’s DICOM file generated two samples per exam. Following import, bone tissue was segmented using standardized, exam-specific thresholds. The STL files of the alveolar ridge and prosthetic plan were then imported and registered to the segmented bone using fiducial landmarks, following the software’s guidelines.

**Fig. 2:**
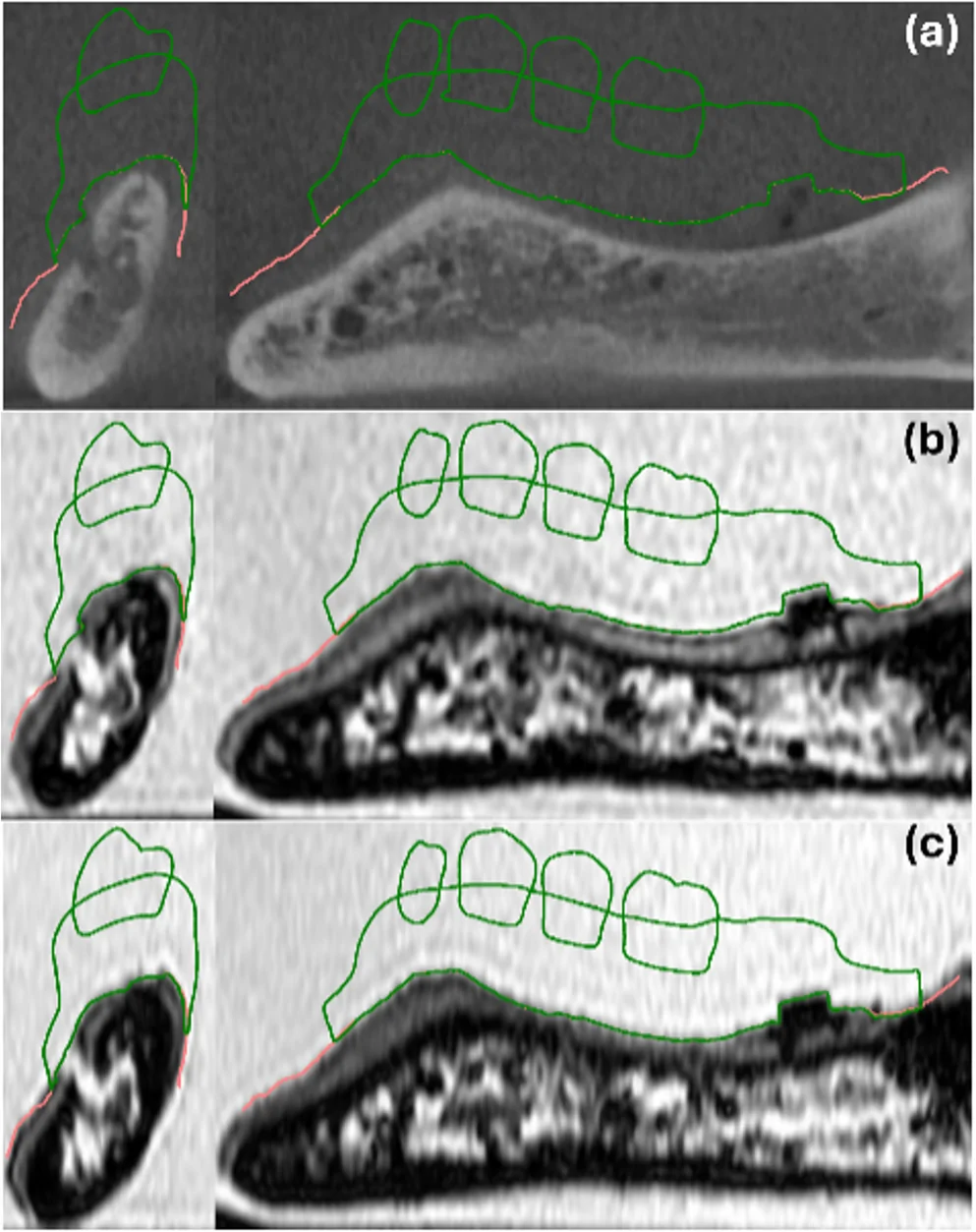
Samples. (**a**) CBCT. (**b**) Anisotropic MRI. (**c**) Isotropic MRI

To ensure consistency, a single investigator standardized the sample construction workflow for all samples, taking into account the patient coordinate system, the panoramic curve, and the color schemes of the segmentation and STL files (Fig. 2). Additionally, all datasets were coded to maintain the anonymity of the exams for the examiners.

### Examiners

Two experienced examiners, with 25 and 8 years of experience in digital implant planning respectively, were blinded to the study details and were calibrated to the proposed workflow.

### Dental implant planning

For each sample, dental implant planning involved tracing the mandibular nerve canal and virtually placing four implants (Bone Level X, 3.75 × 8 mm, Straumann, Switzerland) between the mental foramens. Implant positioning followed the manufacturer’s guidelines: implant angles did not exceed 30 degrees; more than 2 mm was maintained from the mandibular canal, at least 1.5 mm from the buccal and lingual cortical bones, and a minimum of 3 mm between implant platforms.

### Diagnostic accuracy

The implant angular deviation was determined by exporting cylindrical analogs of the selected implants from the planning software in STL format and importing them into metrology software (ZEISS INSPECT Optical 3D, V. 2018, ZEISS, Germany). The position of the occlusal plane was defined as a cylindrical mesh relative to the prosthetic plan. Additionally, geometric elements such as circles and cylinders were created on the implant analogs to serve as reference points for measuring the angle between the longitudinal axes of the implants and the occlusal plane. Implants placed in the incisor and lateral regions were classified as ‘anterior’, while those placed in the canine and premolar regions were classified as ‘posterior’.

**Fig. 3:**
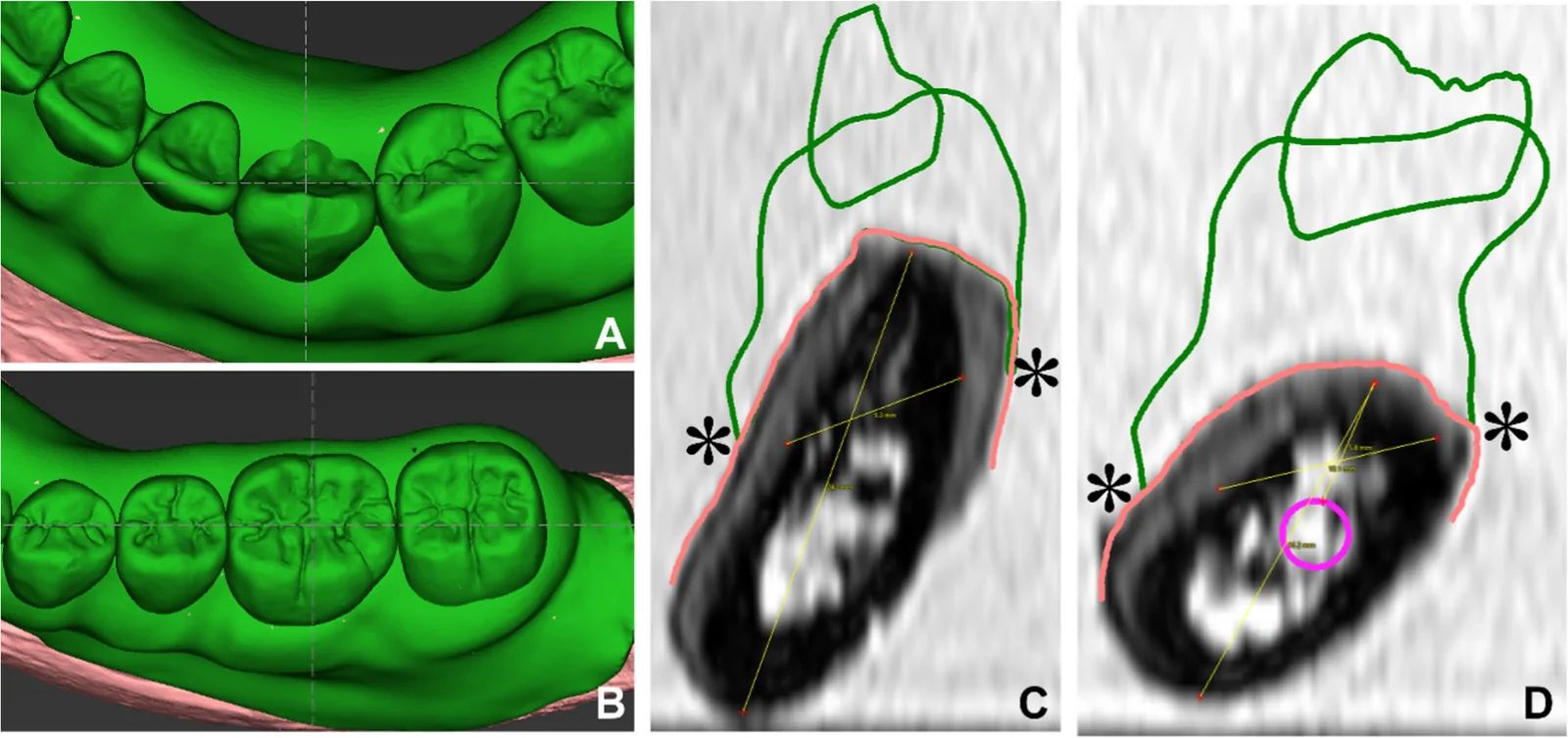
Bone volume measurement with isotropic MRI. (**A**, **B**) Slice selection with prosthetic planning reference. (**C**, **D**) Canine and first molar region bone volume measurements. The asterisk (*) represents the vertical reference point of the prosthetic planning flanges. The purple circle represents the mandibular canal

The examiners used dental implant planning software (coDiagnostiX) to record linear measurements (in millimeters) of the alveolar ridge height, ridge width, and the distance from the ridge to the mandibular nerve canal. To standardize slice selection for these measurements, the software’s “localizer” tool was used to align the measurement plane with the center of the occlusal surface of the canine and first molar in the prosthetic plan (Fig. 3A, B).

The measurements were defined as follows (Fig. 3C, D):

- Alveolar ridge height: The vertical distance between the upper central point of the alveolar ridge crest and the lower central point of the lower border of the mandible.
- Alveolar ridge width: The horizontal distance between the buccal cortical bone and the lingual cortical bone at the lower limit of the buccal and lingual flanges of the prosthetic plan.
- Distance from the ridge to the mandibular nerve canal: The distance from the upper central point of the alveolar bone crest to the upper cortical bone of the mandibular canal.

### Image quality

The examiners assessed the image quality of five anatomical structures (i.e., cortical bone, trabecular bone, alveolar crest, mental foramen, and mandibular canal) using a 5-point Likert scale defined as follows: 1 = visualization of the anatomical structure is not possible, 2 = less than 25% of the anatomical structure is visible, 3 = 25–50% of the anatomical structure is visible, 4 = 50–75% of the anatomical structure is visible, and 5 = complete visualization (100%) of the anatomical structure is possible. For clinical validity, scores were grouped as not valid (score ≤ 2) or valid for clinical use (score ≥ 3) [26]. Additionally, the examiners were able to adjust image settings such as magnification, brightness, and contrast.

### Cost-to-planning-time ratio

The average direct costs of imaging modality (i.e., CBCT and MRI) charged to health insurance/patients in 2024 at the Department of Cranio-Maxillofacial Surgery, University Hospital Muenster, Germany, were recorded in euros. The time required for evaluating the DICOM file, tracking both the right and left mandibular nerve canals, and defining the position of the four implants was recorded by the examiners for each sample using a stopwatch, measured in minutes and seconds. The cost-to-planning-time ratio was calculated individually for each sample as the imaging cost divided by the planning time required for that sample (€/min) [22] .

### Statistical analysis

Angular deviation, linear measurements, planning time, and cost-to-planning-time ratio were assessed for normality using the Shapiro–Wilk test and for variance using Levene’s test. Descriptive statistics were presented as median and interquartile range (IQR). Differences between exams were analyzed using Friedman’s test, followed by Wilcoxon’s signed-rank test for pairwise comparisons. Interexaminer reliability for angular deviation and linear measurements was evaluated using the intraclass correlation coefficient (ICC), which was categorized as weak (≤ 0.20), reasonable (0.21–0.40), moderate (0.41–0.60), good (0.61–0.80), or very good (0.81–1.00) [27]. Image quality was reported as percentage agreement, and significant differences between exams were assessed using the chi-square (χ²) test. All analyses were conducted at a significant level of α = 0.05 (SPSS 26, IBM).

## Results

A total of 96 dental implants were planned, with 32 implants in each of the three exams (i.e., CBCT, anisotropic-MRI, and isotropic-MRI).

### Diagnostic accuracy

Interexaminer reliability for angular deviation varied depending on the anatomical region and exam (Table 2). Implants planned in the anterior region exhibited higher ICC values, ranging from moderate for isotropic to very good for anisotropic and CBCT. In the posterior region, CBCT demonstrated reasonable ICC, while both MRI showed weak correlation. Because the anatomical region and exam influenced interexaminer reliability differently, data from each examiner were analyzed separately. This analysis revealed no significant differences between exam for examiner #1 in either the anterior region (*p* = 0.607) or the posterior region (*p* = 0.687), nor for examiner #2 in the anterior region (*p* = 0.197) or posterior region (*p* = 0.417) (Table 3).

Interexaminer reliability for linear measurements was mostly very good (ICC > 0.827), with two exceptions: good reliability for posterior ridge width using anisotropic (ICC = 0.780) and for the distance from the alveolar ridge to the mandibular nerve canal using isotropic (ICC = 0.783). Because the anatomical region and exam had a similar influence on interexaminer reliability, data from both examiners were analyzed together. This analysis revealed no significant differences between exams for any of the linear measurements (*p* > 0.305) (Table 4).

**Table 2:** Interexaminer reliability of dental implant angular deviation

Anatomical region	Exam	ICC	ICC classification
Anterior	CBCT	0.948	Very good
	Anisotropic	0.866	Very good
	Isotropic	0.584	Moderate
Posterior	CBCT	0.295	Reasonable
	Anisotropic	− 0.154	Weak
	Isotropic	− 0.304	Weak

*CBCT* cone beam computed tomography, *ICC* intraclass correlation coefficient

**Table 3:** Descriptive statistics and comparisons of dental implant angular deviation between imaging examinations, according to each examiner and anatomical region

Anatomical region	Examiner	Exam	Median	IQR	Friedman
Anterior	#1	CBCT	2.79	6.43	0.607
		Anisotropic	4.51	4.88	
		Isotropic	4.39	6.39	
	#2	CBCT	4.23	6.78	0.197
		Anisotropic	6.64	5.87	
		Isotropic	6.80	7.02	
Posterior	#1	CBCT	15.42	10.30	0.687
		Anisotropic	16.38	8.44	
		Isotropic	15.67	6.48	
	#2	CBCT	13.93	4.34	0.417
		Anisotropic	13.90	8.84	
		Isotropic	10.49	5.82	

*CBCT* cone beam computed tomography, *IQR *interquartile range

**Table 4:** Interexaminer reliability for bone volume measurement

Measurement		Exam	ICC	ICC classification	Median	IQR	Friedman
Ridge height	Anterior region	CBCT	0.972	Very good	21.10	7.90	0.305
		Anisotropic	0.937	Very good	21.85	7.68	
		Isotropic	0.969	Very good	22.45	7.18	
	Posterior region	CBCT	0.957	Very good	17.20	6.03	0.717
		Anisotropic	0.931	Very good	18.80	5.82	
		Isotropic	0.964	Very good	17.60	5.33	
Ridge width	Anterior region	CBCT	0.920	Very good	10.20	3.12	0.811
		Anisotropic	0.949	Very good	9.85	3.30	
		Isotropic	0.968	Very good	9.50	3.40	
	Posterior region	CBCT	0.827	Very good	10.00	1.85	0.646
		Anisotropic	0.780	Good	9.65	1.72	
		Isotropic	0.969	Very good	10.05	2.60	
Distance alveolar ridge-mandibular nerve canal		CBCT	0.837	Very good	6.30	5.73	0.740
		Anisotropic	0.971	Very good	5.45	5.65	
		Isotropic	0.783	Good	6.70	4.78	

*CBCT* cone beam computed tomography, *ICC* intraclass correlation coefficient

### Image quality

CBCT consistently demonstrated high interexaminer agreement (> 85%) for most anatomical structures, except for the mandibular nerve canal, which showed lower value (60%). Anisotropic protocol exhibited more variable agreement (ranging from 50% to 100%), with the lowest agreement observed for trabecular bone and the mandibular nerve canal. Isotropic protocol showed moderate agreement across all structures (50–75%), with the lowest agreement for the alveolar ridge and mandibular canal (Table 5).

Although there were no significant differences in the image quality of anatomical structures across the different exam (*p* > 0.132), a tendency toward images not valid for clinical use was observed for the mandibular nerve canal.

**Table 5:** Image quality according to the visualization of anatomical structures

Anatomical structure	Exam	Interexaminer agreement (%)	Diagnostic validity		Group comparisons χ^2^ Test (*p* value)
			Nonvalid for clinical use (%)	Valid for clinical use (%)	
Cortical bone	CBCT	100	0	100	0.344
	Anisotropic	75	12.5	87.5	
	Isotropic	75	12.5	87.5	
Trabecular bone	CBCT	100	0	100	0.132
	Anisotropic	50	25	75	
	Isotropic	75	12.5	87.5	
Mental foramen	CBCT	85	5	95	0.587
	Anisotropic	100	0	100	
	Isotropic	75	12.5	87.5	
Alveolar ridge	CBCT	100	0	100	0.132
	Anisotropic	75	12.5	87.5	
	Isotropic	50	25	75	
Mandibular nerve canal	CBCT	60	20	80	0.270
	Anisotropic	50	50	50	
	Isotropic	50	37.5	62.5	

*CBCT *cone beam computed tomography, *χ*^2^ chi-squared

### Cost-to-planning-time ratio

The cost for each CBCT scan was 180 euros, whereas each MRI scan cost 350 euros. The median planning time was 6.05 min for CBCT, 4.43 min for anisotropic, and 4.35 min for isotropic, with no significant differences between exams (*p* = 0.093) (Table 6). The cost-to-planning-time ratio was significantly lower for CBCT than for anisotropic and isotropic (22.55, 78.92, and 80.46, respectively; *p* = 0.012 for both), and there was no difference between anisotropic and isotropic (*p* = 1.000).

**Table 6:** Descriptive statistics and comparisons for planning time and cost-to-planning-time ratio

Exam	Planning time		Friedman	Cost-to-planning-time ratio (€/min)		Friedman	Wilcoxon		
	Median	IQR	CBCT-Anisotropic-Isotropic	Median	IQR	CBCT- Anisotropic-Isotropic	CBCT-Anisotropic	CBCT-Isotropic	Anisotropic-Isotropic
CBCT	6.05	4.68	0.093	22.55	6.49	0.002*	0.012*	0.012*	1.000
Anisotropic	4.43	1.94		78.92	25.37				
Isotropic	4.35	4.29		80.46	60.61				

*CBCT *cone beam computed tomography

## Discussion

Currently, CBCT is the reference standard imaging exam for dental implant planning, but MRI has shown promising potential, especially for soft tissue visualization and absence of radiation. However, there is still a lack of studies and clinical protocols on the use of MRI in edentulous patients, especially regarding the ideal resolution for dental implant planning. In this study, there were no significant differences between MRI and CBCT regarding diagnostic accuracy, image quality, or planning time, but CBCT was found to be significantly lower cost-to-planning-time ratio, with MRI showing more variability in examiner agreement and lower for image quality.

The amount of literature evaluating dental implant planning using the same MRI sequence as in the present study is limited. One clinical study used the FFE sequence with a larger voxel size (0.70 mm³) and reported that 54.2% of single implant plans were considered ideally positioned, 30% were considered improved, and 15% were considered unacceptable [11]. The authors also noted a low signal-to-noise ratio and poor image contrast, which limited clinical utility. Consequently, the study focused on Turbo-Spin-Echo sequences and reported lower levels of planned implant angular deviation (3 ± 1.15°) compared to CBCT planning.

In the present study, interexaminer reliability for angular deviation was markedly lower in the posterior region. Additionally, greater angular deviation was observed in the posterior region, which is consistent with our previous study utilizing the same methodology but comparing T1- and T2-weighted MRI with CBCT images [12]. Increased angular deviation in the posterior region for all exams was associated with significantly resorbed alveolar ridges in the phantoms [12]. Nonetheless, examiners were instructed to limit implant angulation to within 30° relative to the occlusal plane, in accordance with clinical recommendations for edentulous implant-supported rehabilitations [28]. Thus, the observed angular deviation in all exams remained within this clinical guideline. Moreover, no significant differences in angular deviation were found across anatomical regions or exams, in contrast with our earlier study that showed significant differences for anterior region implants [12]. Differences between studies may be related to the higher resolution and larger sample size of the present study, as well as to the greater anatomical variability offered by the use of three phantoms, instead of just one as in the previous study.

When comparing different imaging modalities (e.g., MRI vs. CBCT), discrepancies in voxel size can impact measurement accuracy, as larger differences can lead to over- or underestimation due to subvoxel size effects [29]. In our study, anisotropic protocol had a finer in-plane resolution (x- and y-dimensions) than isotropic, but a greater slice thickness (z-dimension). As a result, the isotropic protocol had a smaller voxel volume (0.027 mm³) than the anisotropic protocol (0.031 mm³), whereas CBCT had the smallest (0.016 mm³), potentially resulting in more precise representation of smaller phantom sections and affecting measurements. However, no significant differences between imaging methods were observed for linear measurements. This finding aligns with a clinical study comparing implant planning and bone height measurements using proton density as well as T1- and T2-weighted MRI versus CT in 30 patients with single-tooth replacements or partial edentulism, where minimal measurement deviation (0.04 to 1.1 mm) and no significant differences were reported [30]. Conversely, this contrasts with our previous study, which found significant differences between T1-weighted FFE and CBCT for alveolar ridge width in the anterior (*p* = 0.026) and posterior (*p* = 0.028) regions, as well as for the distance from the alveolar ridge to the mandibular nerve canal (*p* = 0.027) [25]. As with angular deviation, these discrepancies may be attributed to the higher resolution, larger sample size, and anatomical variations in the current study.

Although no significant differences were observed in image quality, interexaminer agreement varied across all exams, including CBCT for the mandibular canal, which may have contributed to the lower agreement for anisotropic and isotropic protocols. Furthermore, all exams tended to have poorer visualization of the mandibular nerve canal, likely due to the use of defleshed, dry bones in the phantoms. This observation is consistent with a study using 3 Tesla MRI for digital implant planning and subsequent prosthetic treatment in nine patients [31]. That study reported that cortical and trabecular bone, teeth, and maxillary sinuses were clearly visible on T1-weighted images, whereas the mandibular nerve canal’s visibility was limited. Similarly, previous research demonstrated a tendency for better visualization of cortical and trabecular bone in the mandible and maxilla than for the mandibular nerve canal and mental foramen using T1-weighted FFE at 3 Tesla in patients [18]. Our earlier study also found a clear tendency for poor visualization of the mandibular nerve canal with T1-weighted FFE [25].

Although there was no significant difference in planning time between exams, the cost-to-planning-time ratio was significantly lower for CBCT than for anisotropic and isotropic protocols. The MRI cost in our institution was nearly double that of CBCT when considering only the direct cost to the patient. Installation, maintenance, and personnel costs were not included in this evaluation. The higher cost of MRI scanners can be attributed to the infrastructure needed to prevent accidents and the specialized staff required for operation [32]. However, a dedicated low-magnetic-field dental MRI scanner and operating system could help reduce these costs [32].

It is also important to note that scanning with anisotropic protocol took three times less time (three minutes) than scanning with isotropic (nine minutes). In a clinical setting, longer acquisitions increase the risk of motion artifacts. Since the two protocols did not differ in diagnostic accuracy, image quality, or cost-to-planning-time ratio, the more refined isotropic resolution offered no measurable benefit in this study. These results suggest that the anisotropic protocol could be a pragmatic first choice for routine assessment of ridge dimensions, particularly in patients who cannot remain still for long periods. The isotropic protocol could be reserved for cooperative patients when oblique reformatting is required, although it did not improve mandibular canal visualization in the present study. Because canal visualization was limited in all examinations, larger safety margins may be advisable when implants are planned close to this structure. These suggestions require validation in clinical studies.

This study has limitations due to its in vitro design. Real-life clinical complexities, such as patient movement and dynamic physiological processes, were not represented. Despite the use of artificial soft tissue, the mandible phantom lacked important features such as nerves, blood vessels, and bone marrow, which are present in actual clinical scenarios. The absence of marrow cells, which would increase imaging signal, and surrounding anatomical structures likely contributed to a lower signal-to-noise ratio and reduced image quality [33]. Trapped air bubbles were also observed in the trabecular bone, which could have influenced measurements and visibility due to magnetic susceptibility effects and signal artifacts, potentially resulting in pixel misclassification [33, 34]. Additionally, the phantoms did not consider physiological variations such as age, bone density, disease, or inflammation, thereby reducing clinical relevance.

Several aspects of the phantom design further limit clinical translation. The doped water and uniform, 2-mm silicone layer were designed to simulate, in a standardized manner, the relaxation properties of living tissue and the thickness of the oral mucosa; therefore, image contrast, segmentation thresholds, and surface-scan registration may differ slightly in patients.

In addition, because the phantoms were static, the main expected drawback of the longer isotropic acquisition, motion artifacts, could not be assessed. Image quality evaluation also relied on subjective visual assessment rather than quantitative measures such as SNR and contrast-to-noise ratio, both essential for reliable diagnostic imaging. The absence of these quantitative metrics may affect the confidence and accuracy of image interpretation.

Most previous studies that assessed image quality in CBCT and MRI standardized their evaluations by registering the data and selecting the same slice position for the questionnaire-based evaluations. In contrast, the present study evaluated image quality using the entire volumetric DICOM dataset after planning implant positions and performing linear measurements. As a result, examiners completed the questionnaire after interacting with the dataset in a manner similar to real clinical situations. However, because examiners could freely navigate through the slices, their responses may have been based on different image sections. This could have contributed to the observed interexaminer variability.

The cost-to-planning-time ratio analysis did not assess indirect costs, broader economic implications, or the ratio of additional investment per unit reduction in deviation. In addition, crucial analyses for evaluating the adoption of new technologies, such as willingness-to-pay and cost-benefit analyses, were not included [35, 36]. These limitations warrant the cautious interpretation of the results and highlight the necessity of larger, clinically focused studies. Future research should also establish an acceptable threshold for the cost-effectiveness ratio of using MRI to treat edentulous patients [23].

The slow integration of MRI into dental diagnostic imaging may be due to limited awareness among dentists regarding MRI’s capabilities and advantages over other imaging technologies commonly used in clinical practice [32]. Importantly, new applications of MRI in dentistry require additional training for clinicians, including experienced dental radiologists [37]. Furthermore, dental-specific MRI protocols can be customized to focus on the diagnostic tasks routinely performed in clinical practice [32]. Therefore, the advancement and adoption of MRI in dentistry will depend on both greater awareness and continued research.

## Conclusion

Within the limitations of this in vitro study, no statistically significant differences were observed among cone-beam computed tomography, anisotropic MRI, and isotropic MRI regarding implant angular deviation, linear measurements, or image quality. However, these findings should not be interpreted as evidence of equivalence between imaging modalities. In addition, cone-beam computed tomography showed a lower cost-to-planning-time ratio than anisotropic and isotropic magnetic resonance imaging.

## Data Availability

No datasets were generated or analysed during the current study.
